# Failure of diffusion-weighted imaging in intraoperative 3 Tesla MRI to identify hyperacute strokes during glioma surgery

**DOI:** 10.1038/s41598-021-95505-6

**Published:** 2021-08-09

**Authors:** Stefanos Voglis, Aimee Hiller, Anna-Sophie Hofer, Lazar Tosic, Oliver Bozinov, Luca Regli, Carlo Serra

**Affiliations:** 1 Department of Neurosurgery and Clinical Neuroscience Center, University Hospital and University of Zurich, Frauenklinikstrasse 10, 8091 Zurich, Switzerland; 2 Department of Neurosurgery, Canton Hospital St. Gallen, University of St. Gallen Medical School, Rorschacher Strasse 95, 9007 St. Gallen, Switzerland

**Keywords:** Stroke, CNS cancer, Surgical oncology

## Abstract

Intraoperatively acquired diffusion-weighted imaging (DWI) sequences in cranial tumor surgery are used for early detection of ischemic brain injuries, which could result in impaired neurological outcome and their presence might thus influence the neurosurgeon’s decision on further resection. The phenomenon of false-negative DWI findings in intraoperative magnetic resonance imaging (ioMRI) has only been reported in single cases and therefore yet needs to be further analyzed. This retrospective single-center study’s objective was the identification and characterization of false-negative DWI findings in ioMRI with new or enlarged ischemic areas on postoperative MRI (poMRI). Out of 225 cranial tumor surgeries with intraoperative DWI sequences, 16 cases with no additional resection after ioMRI and available in-time poMRI (< 14 days) were identified. Of these, a total of 12 cases showed false-negative DWI in ioMRI (75%). The most frequent tumor types were oligodendrogliomas and glioblastomas (4 each). In 5/12 cases (41.7%), an ischemic area was already present in ioMRI, however, volumetrically increased in poMRI (mean infarct growth + 2.1 cm^3^; 0.48–3.6), whereas 7 cases (58.3%) harbored totally new infarcts on poMRI (mean infarct volume 0.77 cm^3^; 0.05–1.93). With this study we provide the most comprehensive series of false-negative DWI findings in ioMRI that were not followed by additional resection. Our study underlines the limitations of intraoperative DWI sequences for the detection and size-estimation of hyperacute infarction. The awareness of this phenomenon is crucial for any neurosurgeon utilizing ioMRI.

## Introduction

Intraoperative MRI (ioMRI) is nowadays a commonly used tool providing high quality intraoperative imaging for immediate resection control, especially in brain tumor surgery^1–3^. Besides intraoperative resection control, the updated imaging sets are utilized for further navigation and for the immediate detection of surgical complications^1,4–6^.

Diffusion-weighted imaging (DWI) sequences are routinely used for detection of ischemic infarcts, which can be delineated on DWI sequences within minutes after the ischemic event^7–10^. In ioMRI, DWI sequences are employed for the early detection of new surgery related infarcts potentially impairing neurological outcome^11–14^. Various studies have shown that the quality of intraoperatively acquired DWI sequences is sufficient to delineate new surgery-related ischemic lesions^11,15^. Furthermore, the surgeon's decision whether to continue with further tumor resection after ioMRI control has an impact on the development of new or enlarged ischemic areas around the resection cavity^15^.

However, little is known about the occurrence of false-negative DWI signals in the context of hyperacute stroke imaging, especially when acquired intraoperatively. The available literature regarding ioMRI with false-negative DWI is limited to a few case studies^16,17^, whereas in hyperacute stroke imaging false-negative DWI signals are much more frequently described^18–25^.

To further analyse and describe the phenomenon of false-negative DWI findings we used our institutional registry to identify cases in which no additional resection was performed after ioMRI and where the postoperative MRI (poMRI) showed new or volumetrically enlarged infarct zones.

## Methods

### Patients

**Figure 1:**
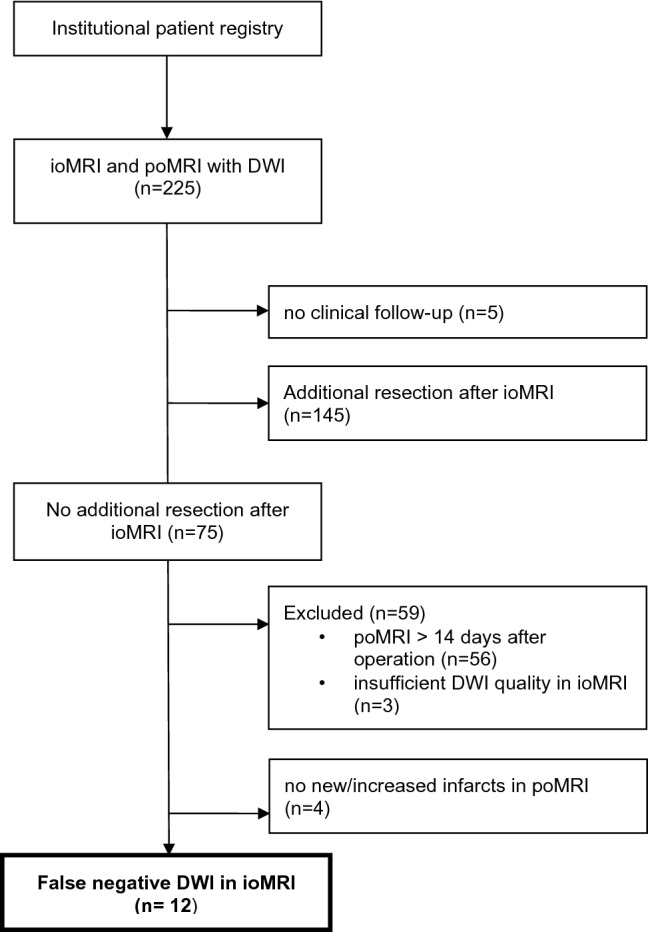
Flowchart of patient inclusion criteria. *ioMRI* intraoperative MRI, *poMRI* postoperative MRI, *DWI* diffusion-weighted imaging.

We retrospectively identified patients who underwent brain tumor surgery with ioMRI (including DWI series and apparent diffusion coefficient (ADC) maps) at the Department of Neurosurgery, University Hospital of Zurich, between January 2013 and October 2019. Cases where no follow-up imaging or clinical examination data were available were excluded (see Fig. 1). For comparability of new infarcts, only patients with poMRI performed within 14 days after surgery were included as it is known that DWI and ADC signal alterations start to normalize around 2 weeks after infarct^26^. Furthermore, all patients were hospitalized with routine vital parameter monitoring until acquisition of poMRI. To specifically identify cases with false-negative DWI imaging, all cases with additional resection after ioMRI were excluded. This information was extracted from the operation reports or by reviewing video records of the surgeries. Additionally—if not clearly stated in the operation report—the microscope video records were reviewed to rule out cases where extensive electrocautery or placement of agents like FLOSEAL for hemostasis were performed after ioMRI. No carmustin wafers were placed in the resection cavity in any of the procedures, neither any other locally applied cytostatic agent was used.

### Intraoperative MRI

Our institution utilizes a 3 Tesla (T) high-field MRI (Siemens 3 T Skyra VD13, Siemens Healthineers, Erlangen, Germany) and a NORAS 8 channel head coil (NORAS MRI products GmbH, Hoechberg, Germany) in a 2-room intraoperative MRI suite concept. After maximum safe tumor resection according to the surgeon's assessment, the dura and skin is closed provisionally, and the patient is prepared for ioMRI transfer. Safety and high quality of intraoperatively acquired MRIs were ensured by following an ioMRI safety checklist that has been published recently^4^. For brain tumor resections, we utilize a standardized ioMRI protocol (including DWI, SWI, as well as multiplanar T1-weighted, T2-weighted and FLAIR image sets). Intraoperative MRI images are immediately evaluated interdisciplinary by the operating surgeon and a board certified neuroradiologist to also consider surgery-specific imaging features (e.g. intentionally left tumor residuals).

### Data collection and ethical considerations

All data were extracted from our patient registry^27^ and by retrospective chart review. The institutional registry and this study have been approved by the local ethical review board (“Kantonale Ethikkommission Zürich”, identifier PB-2017-00093), with a waiver for written informed consent. This study was performed in accordance with the ethical standards of the institutional and local ethics review board and with the 1964 Helsinki declaration and its later amendments or comparable ethical standards.

### Infarct identification and volumetry

Infarcts were determined based on DWI (b0 and b1000 images with b-value 0 and 1000 s/mm^2^, echo time 68 ms, repetition time 6000 ms, slice thickness 4 mm) and ADC image series for each case. Artificial diffusion restrictions due to e.g. microhemorrhage in the resection cavities were ruled out by studying the susceptibility weighted imaging (SWI) sequences. Additionally, immediate surgeon’s feedback was considered by the neuroradiologist to consider any placed hemostatic materials like TABOTAMP or intentionally left tumor remnants which could mimic ischemic-like DWI signal alterations. Cases with insufficient quality to delineate new DWI restrictions in the operation field (e.g. caused by air artifacts) were excluded as well.

Infarcts were only included when adjacent to the resection cavity or the surgical approach and subdivided into 4 classes based on their morphology as described previously^15^: (1) point-shaped infarcts; (2) band-shaped infarcts at the resection cavity; (3) sector-shaped infarcts that involve deeper parts of the parenchyma; (4) territorial infarcts^15^. In the case of multiple isolated infarcts, only the highest class was considered. Volumetry of all infarct volumes was performed semiautomatically based on DWI b100 images using iPlan Net (Brainlab AG, Munich, Germany) “SmartBrush” tool, with manual segmentation adjustments if necessary. No contrast adjustments or other postprocessing methods were used on the original DWI images. The relative infarct volume change (Δ infarct volume; see Table 2) was calculated by subtracting intraoperative from postoperative infarct volumes.

### Statistical analysis

Due to the small case number, we applied descriptive statistics only.

## Results

### Patient characteristics

Based on our retrospective registry review, 225 ioMRI cases with DWI sequences in cranial tumor surgeries were identified. After exclusion of cases not fulfilling inclusion criteria (see Fig. 1), 16 patients were identified where no additional resection was performed after ioMRI and poMRI was acquired in time (see Table 1). Of these, 12 patients showed false-negative ioMRI DWI signals (75%). These cases showed either enlarged or completely new infarcted brain tissue on poMRI. See Table 1 for general patient characteristics: 7/12 cases were female (58%), the mean age was 55.8 years (28–74 years). Mean time until acquisition of intraoperative DWI sequences from surgery start was 172 min (see Supplementary Table 1). Acquisition of ioMRI images took an average of nearly 40 min (mean = 39.9 min, see Supplementary Table 1 for distinct values for each case) without the time for preparation and transfer. Postoperative MRI was conducted on postoperative day 2 to 13 (median: day 2; see Supplementary Table 1). The most frequent histological entities were oligodendroglioma (n = 4, WHO grade II and III) and glioblastoma (n = 4), followed by astrocytoma (n = 1), anaplastic astrocytoma (n = 2), and oligoastrocytoma (n = 1; diagnosis made before publication of revised CNS WHO classification in 2016). Five cases comprised secondary surgeries on recurrent lesions (42%; WHO grade III oligodendrogliomas and glioblastomas), the remaining cases comprised first-time surgeries on the respective tumors. Most tumors were located in the temporal lobe (4/12, 33%), followed by the limbic system (2 hippocampal, 1 in cingulate gyrus), frontal lobe (2), perirolandic region (1), and insula (1).

**Table 1:** Case series characteristics.

#	Age	Sex	Recurrent surgery	Histology	WHO grade	Localization
1	71	f	No	Oligodendroglioma	II	Temporal—T3
2	43	f	Yes	Oligodendroglioma	III	Perirolandic
3	74	f	Yes	Anaplastic astrocytoma	III	Frontal—F1
4	38	f	No	Astrocytoma	II	Limbic—cingulate gyrus
5	68	f	Yes	Glioblastoma	IV	Temporal—fusiform gyrus
6	36	m	Yes	Oligodendroglioma	III	Temporal—T3
7	65	m	No	Glioblastoma	IV	Frontal—F3
8	63	m	No	Anaplastic astrocytoma	III	Temporal—multifocal
9	28	m	No	Oligoastrocytoma*	II	Frontal—F2
10	54	f	No	Glioblastoma	IV	Limbic—hippocampus
11	60	m	No	Glioblastoma	IV	Limbic—cingulate gyrus
12	69	f	Yes	Oligodendroglioma	III	Insular—multifocal

# patient number, *f* female, *m* male, *WHO* World Health Organization, *CNS* central nervous system, *T3* inferior temporal gyrus, *F1* superior frontal gyrus, *F3* inferior frontal gyrus, *F2* middle frontal gyrus.

*Resection and histological diagnosis before revised 2016 CNS WHO classification.

### Infarct characteristics

Five patients (41.7%) showed DWI restrictions already in ioMRI (n = 3 band-shaped; n = 2 point-shaped) with a mean infarct volume of 0.75 ± 0.4 cm^3^ (0.24—1.34; see Table 2). In their poMRI, the DWI sequences showed enlarged infarcted tissue volumes and the infarct class also had changed to the next higher class in all but one patient. Mean infarct volume growth in these 5 cases was 2.1 ± 1.3 cm^3^ (0.48–3.6). Seven patients (58.3%) harbored new infarcts in poMRI without DWI restrictions in previous ioMRI. Infarct classes ranged from point-shaped to sector-shaped, and volumes ranged from 0.05 to 1.93 cm^3^ (mean infarct volume 0.77 ± 0.66 cm^3^). Overall, middle cerebral artery (MCA) vascular territory was affected by ischemia most frequently (9/12, 75%), followed by posterior cerebral artery (PCA) territory (4/12, 33%), and anterior cerebral artery territory (1/12, 8%). Two cases harbored infarcts in both MCA and PCA territory.

**Table 2:** Case series infarct and clinical outcome characteristics.

#	Vasc. territory	Highest class ioMRI	Infarct volume ioMRI	Highest class poMRI	Infarct volume poMRI	Δ Infarct volume	New deficit	Recovery at FU
1	MCA	Band-shaped	1.1	Sector-shaped	3.6	2.5	–	–
2	MCA	Point-shaped	0.52	Sector-shaped	1.27	0.75	Distal arm paresis	Partially
3	MCA	–	–	Point-shaped	0.05	0.05	–	–
4	ACA	–	–	Sector-shaped	1.93	1.93	Arm paresis, hypesthesia	Completely
5	PCA	–	–	Point-shaped	0.38	0.38	–	–
6	PCA	–	–	Band-shaped	1.32	1.32	Sensory aphasia	Completely
7	MCA	–	–	Sector-shaped	0.15	0.15	–	–
8	MCA	Band-shaped	1.34	Sector-shaped	4.94	3.6	–	–
9	MCA	–	–	Band-shaped	0.38	0.38	–	–
10	MCA, PCA	–	–	Sector-shaped	1.19	1.19	Mixed aphasia	Partially
11	MCA, ACA	Point-shaped	0.24	Band-shaped	3.6	3.36	Hemineglect	Partially
12	MCA	Band-shaped	0.57	Band-shaped	1.05	0.48	–	–

Infarct volumes in cm^3^. All pre- and postoperative infarcts were adjacent to the resection cavity. Remote infarcts with no spatial relationship to the surgery or the approach were not considered.

# patient number, *Vas.* vascular, *MCA* middle cerebral artery, *ACA* anterior cerebral artery, *PCA* posterior cerebral artery, *ioMRI* intraoperative MRI, *poMRI* postoperative MRI, *Δ* delta, *FU* follow-up.

Five of 12 patients harbored new neurological deficits postoperatively ranging from mild paresis or hypesthesia to aphasia (see Table 2). The impairments were transient in most cases, and 4/5 patients showed partially whereas 3/5 showed complete recovery at follow-up consultations. Patients with new neurological impairments tended to have larger relative infarct volume changes postoperatively. However, patient #8 (largest infarct growth; Δ infarct volume + 3.6 cm^3^) and patient #1 (third largest infarct growth; Δ infarct volume + 2.5 cm^3^) did not exhibit any new neurological deficits in the postoperative course.

### Illustrative case examples

#### Case 1 (see Fig. 2a)

71-year old female (patient #1) with right temporal (inferior temporal gyrus) WHO grade II oligodendroglioma. IoMRI showed only a very slight band-shaped DWI restriction at the resection cavity. Because ioMRI showed no remaining tumor remnants, no further resection was performed after ioMRI. However, poMRI showed a clear enlargement of the infarcted tissue area, ultimately involving deeper parts of the parenchyma (sector-shaped infarct, relative infarct volume growth + 2.5 cm^3^). However, the patient did not exhibit any new neurological deficits postoperatively.

#### Case 2 (see Fig. 2b)

38-year old female (patient #4) with WHO grade II astrocytoma located in the cingulate gyrus. IoMRI showed no tumor residuals and no obvious signs of DWI restrictions. Discrete DWI hypointensity correlated to blood degradation products in SWI images and was therefore not accounted as an infarct. No additional resection or extensive hemostasis were performed after ioMRI. However, poMRI clearly showed a new sector-shaped infarct of about 1.93 cm^3^. Postoperatively, the patient presented with a new arm paresis and hypesthesia, which revolved completely over time.

**Figure 2:**
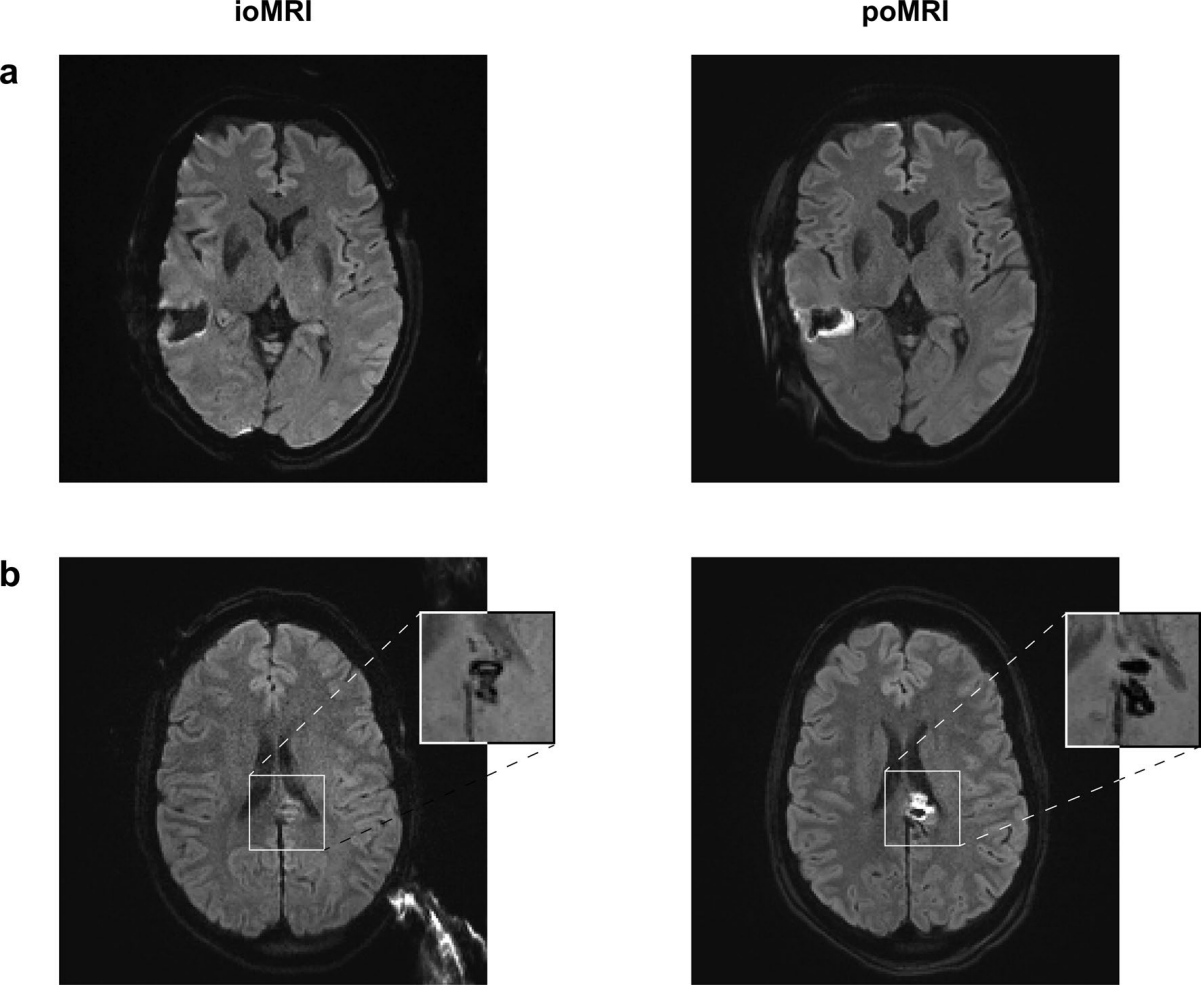
Illustrative case examples. ioMRI (left) and poMRI (right) of two representative cases: (a) Case 1 (patient #1). (b) Case 2 (patient #4). Diffusion-weighted imaging series (a,b) and susceptibility weighted imaging (b, small images). *ioMRI* intraoperative MRI, *poMRI* postoperative MRI.

#### Case 3 (see Fig. 3a)

68-year old female patient (patient #5) with a recurrent right-sided temporal (fusiform gyrus) glioblastoma who underwent re-craniotomy and tumor re-resection. IoMRI showed a total resection and no signs of any DWI restriction. PoMRI, however, depicted a tiny point-shaped DWI lesion with corresponding ADC signal indicating a circumscribed infarct of 0.38 cm^3^. The patient did not show any new postoperative neurological deficits.

#### Case 4 (see Fig. 3b)

**Figure 3:**
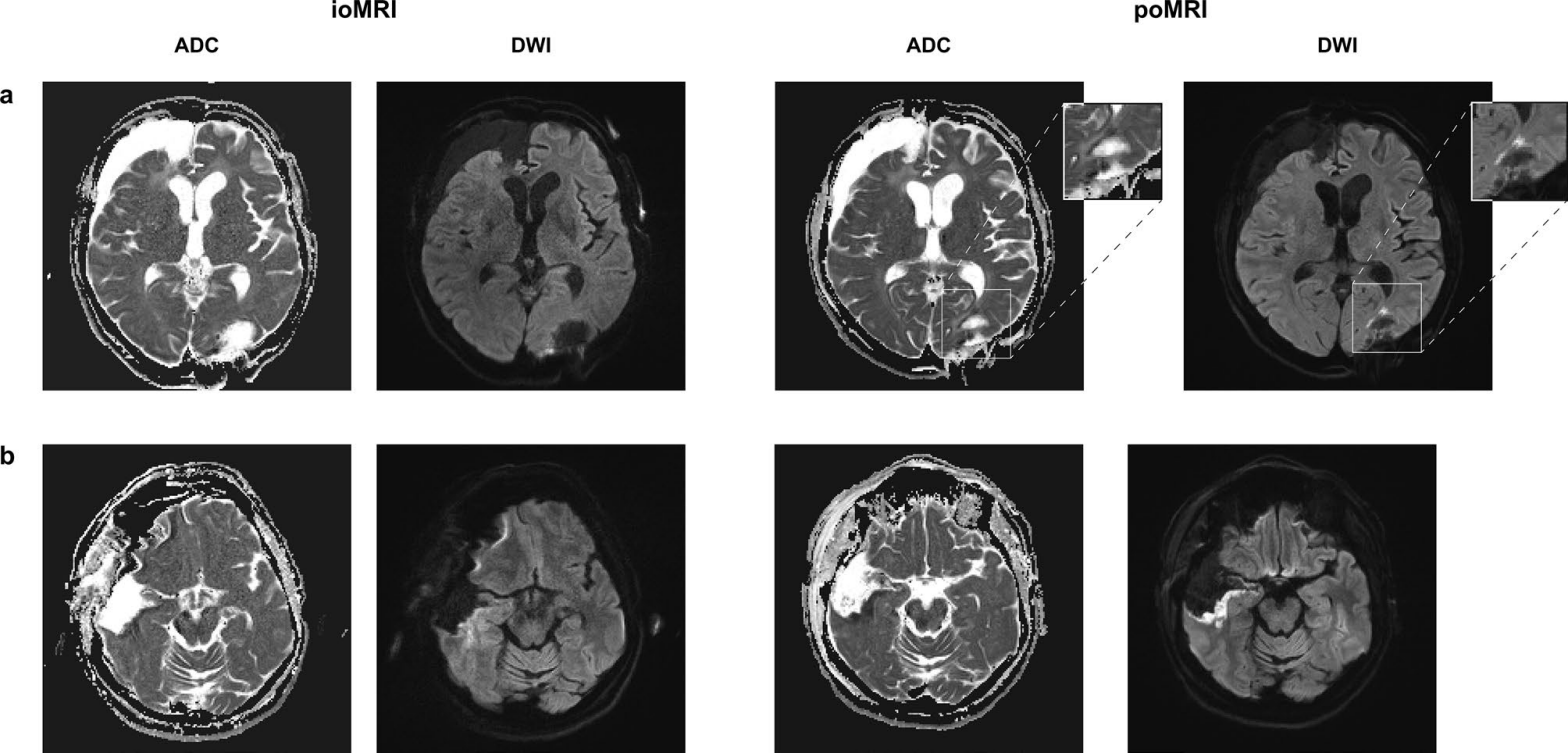
Illustrative case examples. ioMRI (left) and poMRI (right) of two representative cases: (a) Case 3 (patient #5). (b) Case 4 (patient #8). *ioMRI* intraoperative MRI, *poMRI* postoperative MRI, *ADC* apparent diffusion coefficient image, *DWI* diffusion-weighted imaging.

A 63-year old male patient (patient #8) was operated on an anaplastic astrocytoma WHO grade III in the right temporal lobe, spanning multiple gyri. Since there was no residual tumor seen in ioMRI, no further resection was performed. On the third postoperative day, the MRI showed a clear infarct growth compared to the small band-shaped DWI restriction seen intraoperatively (see Fig. 3b, Table 2). Volumetric analysis showed an infarct volume growth of + 3.6 cm^3^. Nevertheless, this increased infarct area was not reflected in an impaired neurological status and the patient remained neurologically asymptomatic.

## Discussion

In the present study we could demonstrate the limitations of ioMRI DWI sequences in to detect hyperacute infarction. Our retrospective analysis revealed a high incidence of false negative intraoperative DWI findings. Screening of 225 ioMRI cases identified 16 patients without additional resection after ioMRI of which 12 harbored enlarged or completely new infarcts on poMRI.

Infarction detection based on DWI sequences in an intraoperative setting is a valuable tool for early diagnosis of surgery related complications. Various groups could show that intraoperatively acquired DWI sequences are of sufficient quality to delineate acute ischemic brain injury^11,15^. Additionally, numerous studies have shown that thin DWI restrictions, at e.g. the resection cavity margin, can be frequently delineated in 20–64% of cases postoperatively after tumor resection^14,15,28,29^. While small ischemic areas around the resection cavity are often considered as “normal postoperative changes”, larger surgically induced infarcts harbor the risk of transient or persistent new neurological deficits.

Despite the reported high sensitivity of DWI sequences to detect ischemic strokes^7,8^, a non-negligible number of articles report cases of hyperacute strokes with initially negative DWI findings^19–25^. This raises the question, whether intraoperatively acquired DWI images underestimate or miss hyperacute or not yet apparent ischemic brain lesions, as our data would also suggest. Oppenheim et al.^20^ retrospectively analyzed 139 stroke patients and found false-negative DWI images in initial MRI scans in 5.8%. The majority of these DWI-negative infarcts were in the vertebrobasilar vascular territory and the initial imaging was performed within 24 h of stroke symptom onset.

In the intraoperative setting, however, the occurrence of false-negative DWI imaging in ioMRI is clearly underreported and includes only reports of single cases^16,17^. Prabhu et al.^17^ presented three cases with false-negative DWI in ioMRI during tumor resection, although in one patient additional resection was performed after intraoperative resection control, and no information was provided about the other two cases regarding extended resection. In our current report we explicitly filtered cases where no further resection was performed after ioMRI in order to prevent accidental inclusion of new infarcts induced surgically after ioMRI. Saint-Martin et al.^16^ analyzed hyperacute infarcts delineated in ioMRI during pediatric brain tumor surgery and showed that ioMRI often underestimates the extent of infarcted brain tissue compared to early poMRI. This is in line with the literature^12,30^ and is also suggested by our data.

In 5 of our cases intraoperative DWI findings underestimated the size of ischemic lesions, and in 7 cases ioMRI showed no clear DWI restrictions at all, even though new infarcts were identified in poMRI. This demonstrates that DWI imaging in ioMRI harbors the risk of either underestimating the size or missing the presence of infarcts. However, in our case series, the minority of patients suffered of a new neurological deficit postoperatively, and some of the patients with the largest infarct volumes even remained clinically asymptomatic.

Various possible explanations for the phenomenon of false-negative DWI findings have been postulated. A focal reduction in cerebral blood flow (CBF) below a threshold critical for neuronal function but still sufficient for proper diffusion was suggested^17,31,32^. Numerous studies have tried to determine the critical CBF threshold for survival of the penumbra in focal ischemia^31,33^.

Additionally, postoperative cerebral vasospasm has been described in as a possible complication especially after resection of posterior fossa tumors^34,35^. Mechanical vessel manipulation could cause delayed vasospasms leading to a transformation of the hypoperfused tissue at risk to an actual ischemic infarct area, which could be missed on ioMRI. A reduced cerebral metabolism during anesthesia^36^ could help collateral vascular supply to maintain a sufficient perfusion during surgery, which might then fail after extubation and normalization of cerebral metabolic needs.

Our 5 cases with already present DWI restrictions in ioMRI and enlarged infarcted area in the follow-up MRI are in line with previous reports showing an enlargement of acute ischemic strokes in subsequent MRI studies with a presumed initial perfusion-diffusion mismatch^37,38^. This perfusion-diffusion mismatch area might be at high risk for definitive infarction due to varying e.g. blood pressure in the immediate postoperative setting or during surgery. The time between vascular injury during surgery and the ioMRI might be also influential for the here described phenomenon. However, our study cohort did not reveal any correlation between the timepoint of ioMRI relative to surgery start and the size of the missed infarct.

In contrast to reports of false-negative DWI in hyperacute ischemic stroke diagnostics where this phenomenon was predominantly described in lesions of the posterior circulation and the brainstem^39^, our case series demonstrates underestimated and false-negative DWI lesions in all vascular territories.

Our case series indicates that normal DWI findings in ioMRI do not rule out new or enlarged infarcts postoperatively, even when no additional resection was performed. False-negative DWI signals occur in all vascular territories and can range from thin marginal areas around the resection cavities up to more pronounced sector-shaped infarcts with involvement of deeper brain tissue.

## Conclusion

This is the first large study showing the limitations of ioMRI DWI sequences to detect or volumetrically estimate surgery related ischemic events in brain tumor surgery. However, due to the retrospective design of our analysis and the low case number, generalizable estimates of the actual incidence of false-negative DWI in ioMRI cannot be provided. Additionally, an unequivocal distinction between the etiology for the missed or underestimated infarcts (e.g. DWI sensitivity or surgically related "tissue at risk") is not possible based on this study. Nevertheless, the awareness of this phenomenon is important for any neurosurgeon utilizing ioMRI. Since any intraoperative detection of vascular injury can influence the surgeon’s decision regarding the scope of further resection.

## Supplementary Information

Supplementary Table 1.

## Data Availability

All data is available within this publication.
