# Supplementary material for: Failure of diffusion-weighted imaging in intraoperative 3 Tesla MRI to identify hyperacute strokes during glioma surgery
Source: Sci Rep. 2021 Aug 9;11:16137. doi: 10.1038/s41598-021-95505-6 (PMC8352886; doi:10.1038/s41598-021-95505-6)
Supplement: Supplementary file 1 — Supplementary Table 1. [file 41598_2021_95505_MOESM1_ESM.docx]

| # | postoperative  day of poMRI | Time to  ioMRI [min] | Time in  ioMRI [min] |
| --- | --- | --- | --- |
| 1 | 1 | 142 | 40 |
| 2 | 1 | 188 | 35 |
| 3 | 1 | 137 | 33 |
| 4 | 2 | 197 | 41 |
| 5 | 2 | 113 | 25 |
| 6 | 2 | 43 | 40 |
| 7 | 2 | 70 | 45 |
| 8 | 3 | 191 | 60 |
| 9 | 4 | 168 | 40 |
| 10 | 6 | 283 | 40 |
| 11 | 10 | 317 | 35 |
| 12 | 13 | 219 | 45 |
| Σ: | median = 2 | mean = 172.3 | mean = 39.9 |

**Supplementary Table 1:** Additional case characteristics regarding MRI usage

Postoperative day on which the poMRI was conducted, time per case from skin incision until intraoperative DWI sequence acquisition and time per case spend in ioMRI

# patient number, poMRI postoperative MRI, ioMRI intraoperative MRI, min minutes
